# Assembling unmapped reads reveals hidden variation in South Asian genomes

**DOI:** 10.1101/2025.05.14.653340

**Published:** 2025-05-14

**Authors:** Arun Das, Arjun Biddanda, Rajiv C. McCoy, Michael C. Schatz

**Affiliations:** 1 Department of Computer Science, Johns Hopkins University, Baltimore, MD, 21218, USA; 2 Department of Biology, Johns Hopkins University, Baltimore, MD 21218, USA

## Abstract

Conventional genome mapping-based approaches systematically miss genetic variation, particularly in regions that substantially differ from the reference. To explore this hidden variation, we examined unmapped and poorly mapped reads from the genomes of 640 human individuals from South Asian (SAS) populations in the 1000 Genomes Project and the Simons Genome Diversity Project. We assembled tens of megabases of non-redundant sequence in tens of thousands of large contigs, much of which is present in both SAS and non-SAS populations. We demonstrated that much of this sequence is not discovered by traditional variant discovery approaches even when using complete genomes and pangenomes. Across 20,000 placed contigs, we found 8,215 intersections with 106 protein coding genes and >15,000 placements within 1 kbp of a known GWAS hit. We used long read data from a subset of samples to validate the majority of their assembled sequences, aligned RNA-seq data to identify hundreds of unplaced contigs with transcriptional potential, and queried existing nucleotide databases to evaluate the origins of the remaining unplaced sequences. Our results highlight the limitations of even the most complete reference genomes and provide a model for understanding the distribution of hidden variation in any human population.

## Introduction

Genome sequencing studies have not uniformly spanned the spectrum of human diversity. As of 2019, >80% of participants in genomic studies were of predominantly European ancestries, with poor representation from other geographic regions ^1,2^. While recent sequencing and assembly efforts are now addressing this gap ^3–5^, the persisting biases hinder fundamental biological research and clinical interpretation ^6,7^.

One such underrepresented region is South Asia, home to more than 1.9 billion people ^8^, with another 35-40 million people in its diaspora ^9–11^. South Asians cumulatively comprise only ~2% of individuals in genome-wide association studies (GWAS), and even within this broader group, certain ethnicities are over- or underrepresented ^1^ (Supplementary Figure 1). Prior efforts to catalog South Asian diversity are largely out-dated, working with genotyping arrays or lower quality sequencing data, or performed using older reference genomes ^12,13^. While there is an abundance of low-coverage sequencing data from individuals of South Asian ancestries, curated, high-coverage, open-access datasets from South Asian samples are lacking. Two notable exceptions are the 1000 Genomes Project (1KGP) and Simons Genome Diversity Project (SGDP), which offer high-coverage whole-genome sequencing data from 640 South Asian individuals across 24 populations.

Even with high-quality data, other biases affect researchers’ ability to analyze genome sequences. Notably, conventional genomic analysis typically involves the use of a reference genome, but a single reference genome may obscure variation in regions poorly represented by the reference. Even for the complete T2T-CHM13 reference genome, insertions relative to the reference and highly divergent haplotypes may fail to represent sequences present in newly sequenced genomes ^3,4,14^. This reference bias was highlighted in the African Pangenome project ^15^, which found hundreds of megabases of sequence present in African individuals that are absent in GRCh38. Similar efforts with other populations have similarly identified tens of megabases of sequence that are missing in widely used reference genomes ^16–18^. Furthermore, even current pangenomes cannot fully overcome this challenge, as human populations possess an excess of ultra-rare variation that will not be adequately captured by any small panel of references ^5,19^.

In this work, we cataloged genetic diversity within South Asian populations and tested the limitations of reference genomes and pangenomes. To this end, we re-analyzed existing short read sequencing data from 640 South Asian individuals across 24 populations in the 1KGP ^20,21^ and SGDP ^22^ datasets and assembled sequences that are present in these samples but absent from the reference genomes. We then validated thousands of large contigs assembled from these unmapped reads through alignment to long-read based assemblies ^23^ We find that approximately 20,000 partially aligned and could thus be placed to a region of T2T-CHM13, facilitating interpretation. These placements include a number of overlaps with biologically relevant regions, including thousands of intersections with over a hundred protein coding genes, and thousands of placements within one kilobase (kb) of known genome-wide association study (GWAS) peaks. In contrast, existing variant finding tools failed to detect and place most of these large contigs and ignored most of the unplaced novel sequence. We also examined the potential functional and phenotypic relevance of this variation by leveraging external RNA-seq, highlighting widespread transcriptional potential. Together, our research highlights the limitations of both linear and pangenome references and presents a generalizable framework and open-source software pipelines for recovering hidden variation from existing sequencing data.

All code and scripts used in this analysis are available at https://github.com/arun96/SouthAsianGenomeDiversity and may be used to study variation in any population.

## Results

### Alignment, assembly, and filtering

#### Alignment and assembly of data from the 1KGP SAS set

We first aligned reads from the 601 1KGP SAS individuals against the T2T-CHM13 v2.0 ^3^ and GRCh38 ^24^ linear references. Alignment to the newer T2T-CHM13 reference improved the alignment rate (+0.7-1.0%) compared to GRCh38, and increased the number of reads concordantly aligned to a single locus (+1.0%). This improvement was expected, since T2T-CHM13 adds hundreds of millions of bases of newly resolved sequence. However, despite this improvement, 1.3-1.7% of all reads per individual remain unaligned against T2T-CHM13. These reads represent an opportunity for discovery, as they will contain novel sequences these individuals possess relative to the chosen reference as well as reads with excessive sequencing errors or other technical artifacts. In addition to extracting unaligned reads against the references, we also investigate a less stringent threshold of poorly aligned reads, defined as reads that align with quality score <20, which resulted in about 20-30x as many extracted reads as the stricter “no alignment” threshold. Results from the alignment and assembly of these reads are provided in the Supplementary Materials.

Unaligned reads were then assembled into larger contigs whose origins could be more easily assessed. Using the MEGAHIT genome assembler ^25^ on reads that failed to align against T2T-CHM13, we initially assembled ~1 Mb of sequence per individual. However, much of this sequence was composed of short contigs, potentially reflecting contamination or assembly errors. Following a process similar to the African Pangenome study ^15^, this set was filtered to exclude contigs <1 kbp in length. This reduced the amount of assembled sequence to approximately 550 kb per individual. These contigs were then screened using BLAST ^26^ and Centrifuge ^27^ against databases of known bacterial and microbial sequences, removing contigs with any matches to these contaminants. This step removed few contigs (< 0.5%), but increased confidence that the remaining sequences were of human origin. Of the contaminant contigs that were removed, the majority matched to adapter sequences or common human contaminants such as herpes virus.

**Figure 2.**
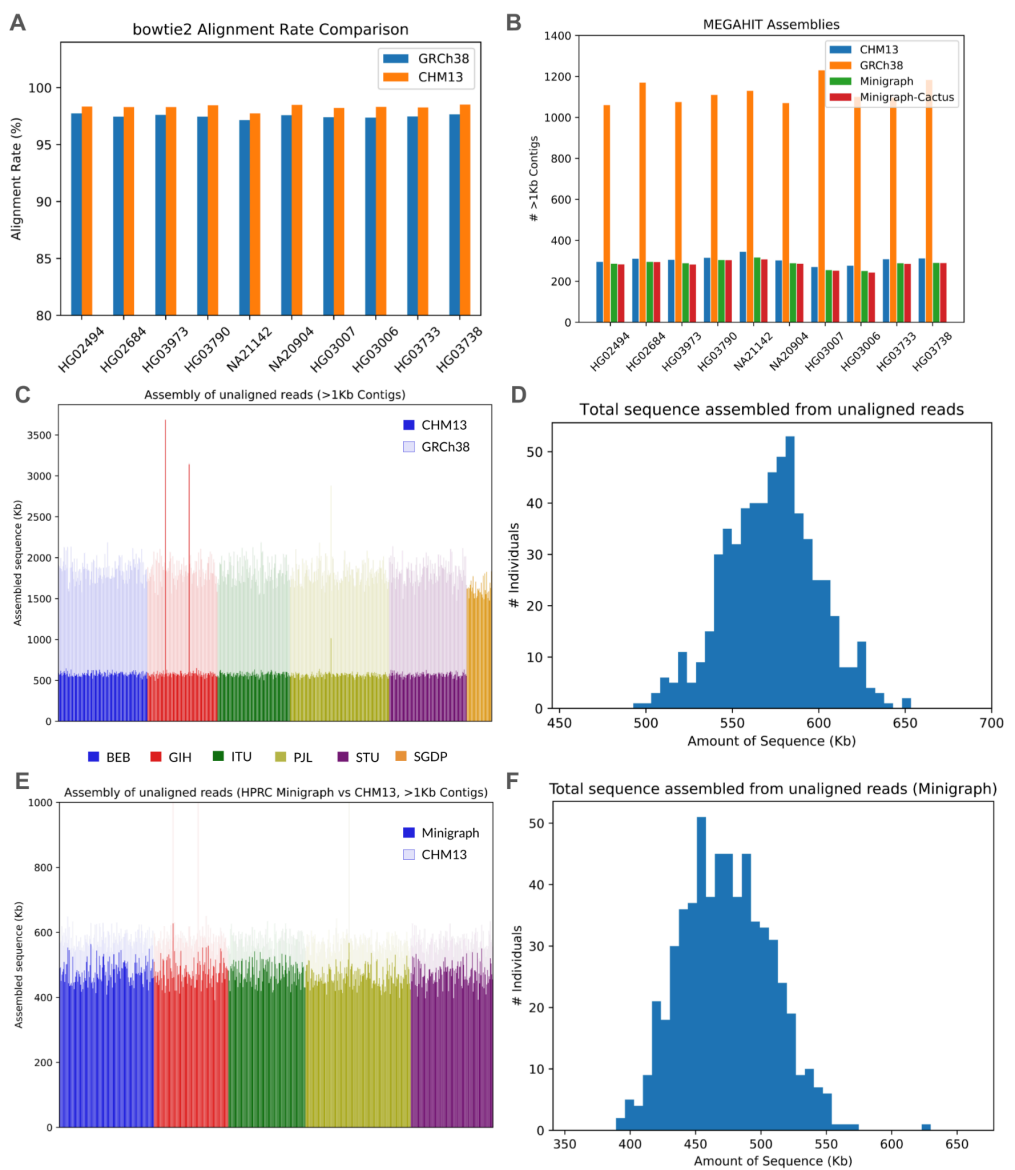
Genome Alignment and Assembly. **(a)** Comparison of the bowtie2 alignment rate between GRCh38 and T2T-CHM13 across 10 SAS samples. **(b)** Comparison of the number of assembled >1 Kb contigs across T2T-CHM13, GRCh38, and the HPRC constructions across the same 10 SAS samples. **(c)** Amount of assembled sequence from unaligned reads against linear references per individual across the entire 1KGP and SGDP SAS sets. For the 1KGP subset, this is against both T2T-CHM13 (solid) and GRCh38 (translucent). **(d)** Distribution of the assembled sequence from unaligned reads against T2T-CHM13 across the 1KGP SAS individuals. **(e)** Amount of assembled sequence from unaligned reads against the Minigraph pangenome reference per individual across the entire 1KGP SAS set. The T2T-CHM13 amounts are translucent and displayed behind for comparison. **(f)** Distribution of the assembled sequence from unaligned reads against the Minigraph pangenome reference across the 1KGP SAS individuals.

After filtering, we assembled an average of ~550 kb of sequence per SAS individual, totaling approximately 350 Mb across the entire 1KGP SAS set (Figure 2c–d). The size of these contigs varied greatly, with an average of 70 contigs >5 kb and 18 contigs >10 kb per individual. The average N50 for these filtered assemblies was 1.95 kb, and the average size of the largest contig assembled per individual was 15-18 kb, with the largest contigs in the entire set spanning over 50 kb. This is a substantial reduction compared to the sequence assembled using the corresponding unaligned reads against GRCh38, where an average of 2 Mb of sequence was assembled per individual (Figure 2c). We observed 3 outliers when assembling unaligned reads against T2T-CHM13 (Figure 2c); however, aligning these contigs against themselves revealed a large amount of nearly duplicated sequence, effectively resolving these outliers.

We further evaluated the impact of the complete T2T-CHM13 reference by performing “cross alignment”, where the contigs assembled from T2T-CHM13 unmapped reads were aligned against those assembled from GRCh38 unmapped reads (Supplementary Figure 3). The T2T-CHM13 contigs are largely a subset of the GRCh38 contigs, with 70% having a high identity, high mapping quality alignment against a GRCh38 contig. Conversely, 75% GRCh38 contigs did not align well to the T2T-CHM13 set, but 70% aligned well to the T2T-CHM13 reference itself, reflecting its completeness (a similar result was observed when aligning unaligned read contigs originally assembled against GRCh38 in the APG study ^15^ against the T2T-CHM13 reference ^4^). Our results suggest that use of GRCh38 obscures potential genetic variation, particularly within erroneously assembled or unresolved regions that are later resolved in T2T-CHM13. For these reasons, unless otherwise noted, we focus our analyses on the contigs assembled from T2T-CHM13-unaligned reads for all subsequent analyses.

#### Alignment and assembly of data from the SGDP SAS set

We repeated the alignment and assembly steps with data from 39 SAS individuals across 19 populations from the SGDP dataset. For 37 out of the 39 individuals, assembly of their unaligned reads against T2T-CHM13 (0.9 - 1.7% of the total input reads) and filtering to >1 kb non-contaminant contigs resulted in 1.4 - 1.8 Mb of assembled sequence per individual. Each assembly contained ~500 contigs with average N50s of 3.2 kb, and the largest assembled sequences reached ~40 kb (Supplementary Figure 4). We attribute the larger span of non-contaminate contigs due to the higher sequencing depth of SGDP compared to 1KGP.

The remaining two individuals are from the Kusunda group, and had markedly different alignment and assembly results. Specifically, these samples exhibited alignment rates ~10% lower than the other 37 SGDP individuals, and their unaligned reads assembled into >200x as much sequence (Supplementary Figure 5). BLAST and Centrifuge did not categorize these sequences as known human sequence or contaminants, and most quality metrics were in line with other SGDP SAS samples. Full details of these two outliers are provided in Supplementary Figure 6, but we hypothesize that the excess sequences originate from a mixture of poorly categorized microbial contaminant and sequencing artifacts, and we therefore excluded these two individuals from subsequent analyses.

#### Pangenomic improvements in alignment and assembly

We repeated the assembly process with unaligned reads against the HPRC Minigraph and Minigraph-Cactus v1.0 pangenome references. The HPRC pangenome references were constructed by augmenting the T2T-CHM13 reference with the genome sequences from 47 individuals, including one individual from the South Asian continental group. Against the Minigraph reference, alignment rates rose 0.3-1.1% per individual, which in some cases halved the number of unaligned reads compared to T2T-CHM13. The expanded Minigraph-Cactus reference further improved the alignment rates, though this improvement was marginal (<0.1%). For the SAS individual also present in the Minigraph and Minigraph-Cactus reference, we observed near perfect alignment across the genome, with the overall alignment rate rising from 98.7% against T2T-CHM13 to 99.8% against the Minigraph reference.

Despite the sizable declines in the number of unaligned reads, the reductions in the amounts of assembled sequence were not as dramatic (Figure 2e). Against the Minigraph-based reference, we assembled 450-500 kb of sequence in >1 kb non-contaminant contigs from unaligned reads per 1KGP SAS individual, a ~10% decrease in sequence compared to the same individuals against T2T-CHM13 (Figure 2e–f). This suggests that many of the newly aligned reads would have assembled into short contigs discarded during our filtering step. The number of >1 kb non-contaminant contigs declined even less (Figure 2b), suggesting parts of previously assembled contigs had been resolved. The average N50 of the filtered assemblies was 1.9 kb and the largest assembled contigs per individual are 15-18 kb (maximum ~50 kb). With the augmented Minigraph-Cactus reference, we observed additional minor decreases in the amount of assembled sequence (<5%) and number of contigs that composed this sequence (<3.5%). These observations were expected, given that the Minigraph-Cactus reference only adds sequences that are a fraction of a read length long.

#### Comparison to non-SAS populations

To contextualize these results, we performed a comparison using samples from non-SAS populations. Specifically, we repeated the same alignment and assembly steps using 10 randomly selected individuals, 5 XX and 5 XY, from each of the 21 non-SAS populations in the 1KGP dataset. Across the two linear references, the results with the non-SAS set were similar to those from the SAS set, with approximately three times as much sequence is assembled per individual against GRCh38 than against T2T-CHM13 from unaligned reads. However, individuals in the SAS set had on average 10-15% less assembled sequence than the individuals in the seven African (AFR) populations, and on average 5% more sequence than the individuals in the non-AFR, non-SAS cohorts (Supplementary Figure 7).

It is worth noting that while T2T-CHM13 exhibits the greatest genetic similarity to 1KGP samples from the European (EUR) continental group, the amount of sequence assembled for samples from EUR populations is nearly the same as any other non-AFR population. This underscores the fact that human genetic diversity tends to be either globally common (present in all populations at relatively similar frequencies) or rare (geographically restricted, but only because it is rare and is also rare within a given population) ^28^. More broadly, these results are consistent with two well established patterns about human genetic variation. First, the diversity in African populations exceeds that in non-African populations as a result of the out-of-Africa bottleneck ^29,30^. Second, there is a substantial amount of sequence across individuals that is not captured by a single reference, reflecting the excess of rare variation in human genomes due to rapid population growth ^31^.

Overall, these results highlight the improvements offered by including sequence from multiple individuals in a pangenome, thus capturing common variation that would have previously remained unmapped and discarded. However, the amount of sequence is still assembled from unaligned reads against these augmented pangenome reference shows how much variation is still being missed.

### Shared sequence analysis

Across the SAS set, we assembled approximately 350 Mb of sequence in filtered contigs from unaligned reads against T2T-CHM13. However, there are redundancies in this assembled sequence, with many contigs that are the same or nearly the same across multiple individuals if those individuals carry the same polymorphism from the reference (Supplementary Figure 8). Therefore, we also attempted to determine whether a sequence is uniquely found in a single individual or shared between multiple individuals by performing an all-vs-all alignment assembled contigs using Minimap2 ^32^. In total, this procedure collapsed the 199,564 contigs totalling 410 Mb of sequence into 13,875 shared contigs and 2,137 unique contigs totalling 47 Mb of sequence (the “non-redundant” set of SAS contigs). The frequency spectrum demonstrates that 14% of contigs are present in <10% of the individuals in the combined SAS set, whereas 1% are present in >90% of the set. (Figure 4e, Supplementary Figure 9–10).

We were also interested in how much of the sequence from these SAS donors is present in genomes of individuals from non-SAS populations. To quantify this proportion, we aligned read data from the selected 210 non-SAS individuals against the non-redundant SAS contigs. We found that, on average, 3.8 - 4.5% of reads from each individual align to this set of contigs, with individuals of all 21 non-SAS populations exhibiting roughly the same alignment rate. This observation further highlights the fact that most variation is rare, and that the variation that is common tends to be shared across all populations.

### Validation with long reads

The initial data release from the 1KGP ONT effort ^23^ contains long read data from 21 individuals present in our SAS set, which can be used to help validate the assembled contigs from these individuals. We constructed simple assemblies for these 21 individuals with their long reads using the Flye assembler ^33^. The assemblies contain 2.81 - 2.84 Gb of sequence, with N50s ranging from 31.9 - 49.2 Mb and mean coverage of 29-47x. Although these assemblies are far from telomere-to-telomere completeness, we observed that 40-50% of these previously unaligned reads aligned well to the new personalized long read assemblies of the respective samples. The “rescue” of these unaligned reads highlights the limitations of a single reference genome and confirms that much of the missing sequence is variation from loci that are easily assembled from long-reads.

We also aligned the assembled >1 kb unaligned read T2T-CHM13 contigs against the long read assemblies. Across the 21 SAS individuals over 85% of their assembled contigs aligned with high identity to their long read genome assembly (Figure 4c). This further confirmed that the vast majority of these contigs are sequences truly found in the human genome. The remaining 15% of contigs probably originate from more complex regions that were not accurately assembled in a single pass and may require more tailored assembly parameters or additional sequencing technologies that better capture these regions.

### Placement of assembled contigs

Placing these assembled contigs back into the reference genome is possible through direct alignment of the contigs to the reference genome or using data from mapped mate pair reads (as used in APG ^15^). Approximately 40% of contigs aligned in some way to the T2T-CHM13 reference, dropping to ~10% when we required alignment lengths of at least 500 bp. This meant that approximately ~350 contigs remained unaligned on average per individual, and these are likely to contain any entirely novel sequence. Alternatively, we can attempt to relate these contigs to the reference based on the mapping status of mate pairs used in the assembly. This method placed fewer contigs than direct alignment (3,952), but addressed cases where a contig diverges from the reference sequence. Combining these two methods, 19,691 of 199,564 contigs (~10%) >1 kb in length were placed against T2T-CHM13. >85% of these placed contigs from the 21 SAS individuals with long read data were validated against their long read assemblies. While extending across all chromosomes, these placements were not evenly distributed throughout the genome, with enrichments noted on chromosomes 6, 9, and Y (Supplementary Figure 11), potentially driven by their higher repeat content (e.g., the HLA locus on chr6).

#### Intersection with annotated elements

**Figure 3.**
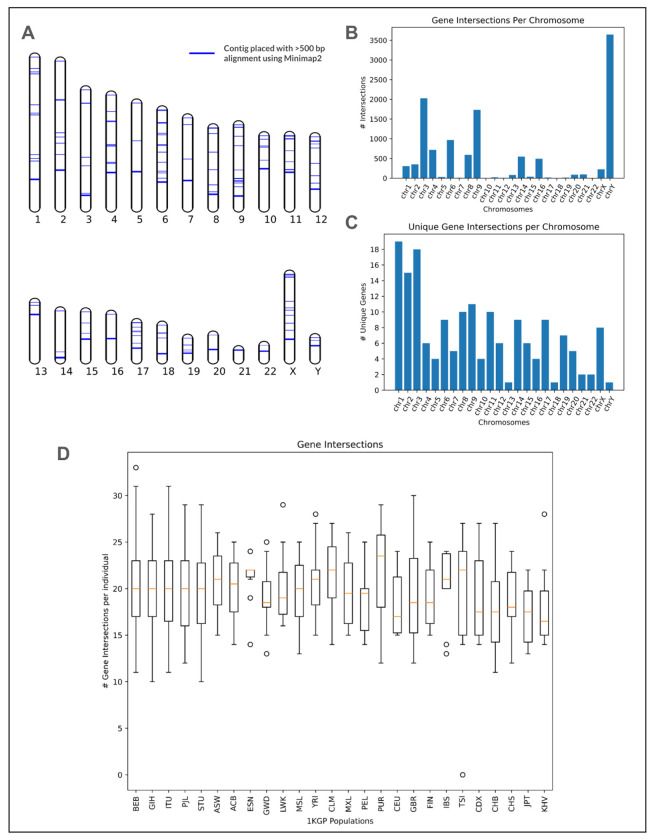
Placements & regions with “hidden” SA variation. **(a)** Visualization of placements against the T2T-CHM13 reference. Each line represents an assembled unaligned read contig with a significant (>500 bp) Minimap2 alignment against the T2T-CHM13 reference. **(b)** Counts of the number of gene elements in T2T-CHM13 v2.0 intersecting placed contigs. **(d)** Distribution of the number of gene intersections per individual across the 26 1KGP populations.

**Figure 4.**
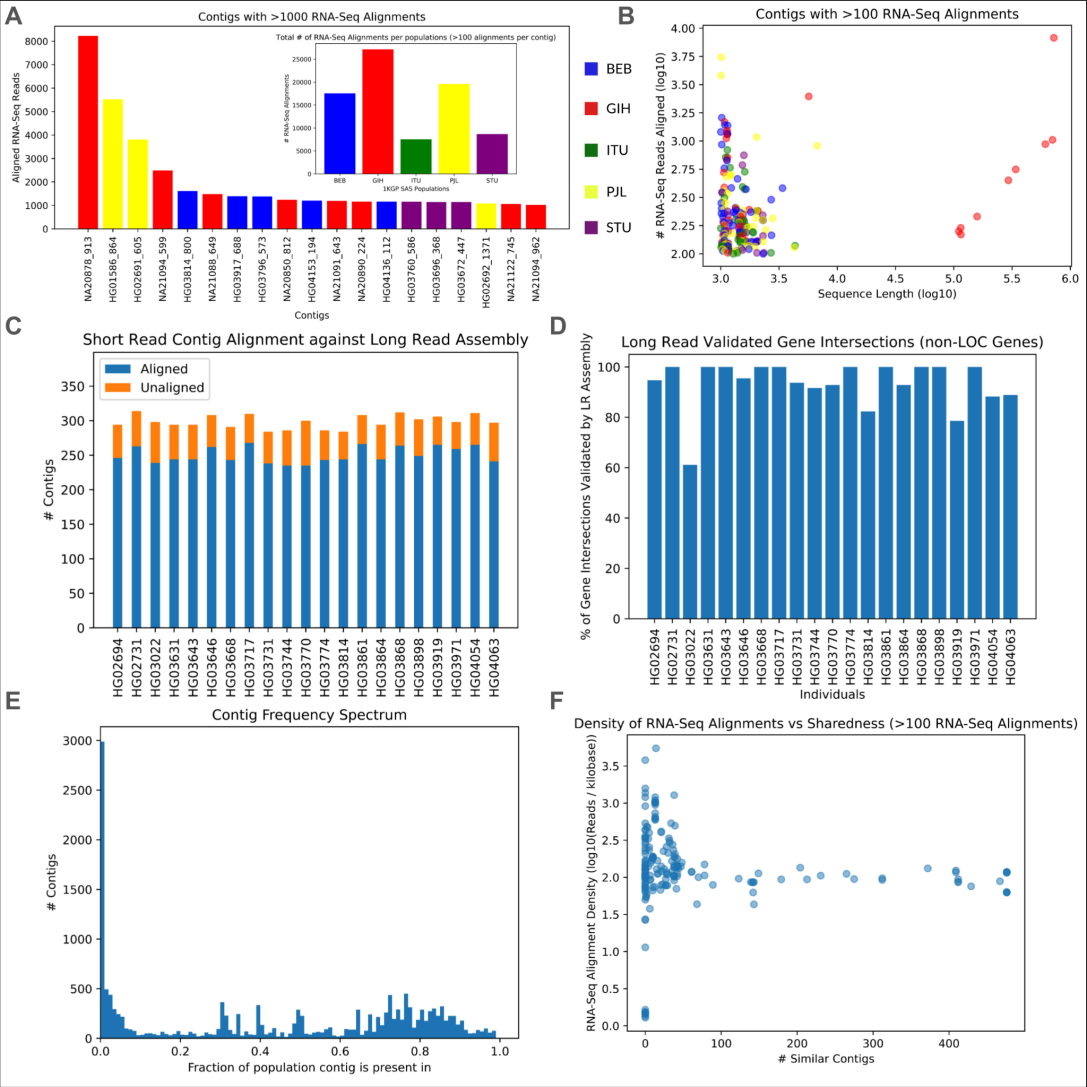
RNA-Seq Alignment, Long Read Validation and characterizing shared sequence. **(a)** The 20 contigs across the 140 SAS individuals with the most RNA-Seq reads aligned to them during the all-vs-all alignment step, colored according to their SAS subpopulation. These contigs come from 19 individuals across the SAS set. Inset in (a) is the number of contigs with >100 RNA-Seq alignments in each of the five SAS sub-populations. **(b)** All contigs with >100 RNA-Seq alignments during all-vs-all alignment, with the number of alignments plotted against their sequence length, colored according to their SAS subpopulation. **(c)** The number of unaligned read contigs in 21 SAS individuals that are successfully aligned against a personalized long read reference from the same individual. **(d)** The fraction of gene intersections in 21 individuals with long read data that are in “long read validated” contigs. **(e)** Distribution of the occurrence of contigs after collapsing shared sequence, plotted in terms of the fraction of the population each collapsed contig appears in. **(f)** The level of sharedness present in the most aligned-to contigs from RNA-Seq analysis, plotted against the density of RNA-seq alignments (number of reads aligned per kilobase of contig, log_10_).

We intersected the placed contigs with the “JHU RefSeqv110 + Liftoff v5.1” gene annotations from the T2T Consortium using bedtools ^34^. Across the SAS set there were 52,727 contig intersections with known transcripts, 12,021 with known genes, 5,050 with known exons, and 3,015 with known CDS regions (Figure 3a). These are not evenly distributed across the genome, both in terms of the total number of intersections (Supplementary Figure 12) and the number of gene intersections (Figure 3b). Counting the number of unique annotated elements, there were contig intersections with 1,189 unique transcripts, 172 genes, 33 exons and 6 CDS regions, and the distribution was more even across the genome (Figure 3c). 136 of the intersected genes are non-LOC and 106 of these have an NCBI gene type of “Protein Coding”. While the majority of the 106 protein coding genes have intersections in just a few individuals, some are more widely intersected (Supplementary Figure 13). Across the 21 individuals in the SAS set with long read sequencing data, we validated 92.7% (331/357) of contig/non-LOC gene intersections (Figure 4d).

Among the gene intersections, 11 intersected genes had connections to eye-related disorders (*AGBL1, CPAMD8, CRB1, HMCN1, KIF14, LIN9, MYO7A, PPEF1, TTC39C, USH2A* and *WDR17*), as well as genes that are linked to thyroid function (*TSHR*), facial dysmorphia (*GPC3, CPA6, OPHN1, OR51A4* and *SNP1*), ciliary function (*CC2D2A, DRC1, NME8, TTC39C*), and several tumor suppressors and cancers (*DLEC1, DMBT1, LIN9, PTPRO, SEM1, SYK and ZNF292*). Full details of each intersected gene are in Supplementary Table 1, and a list of all intersections with annotated elements and genes are in Supplementary Table 6–7 respectively.

#### Analyzing and utilizing potential linkage disequilibrium within the shared contig set

**Figure 5.**
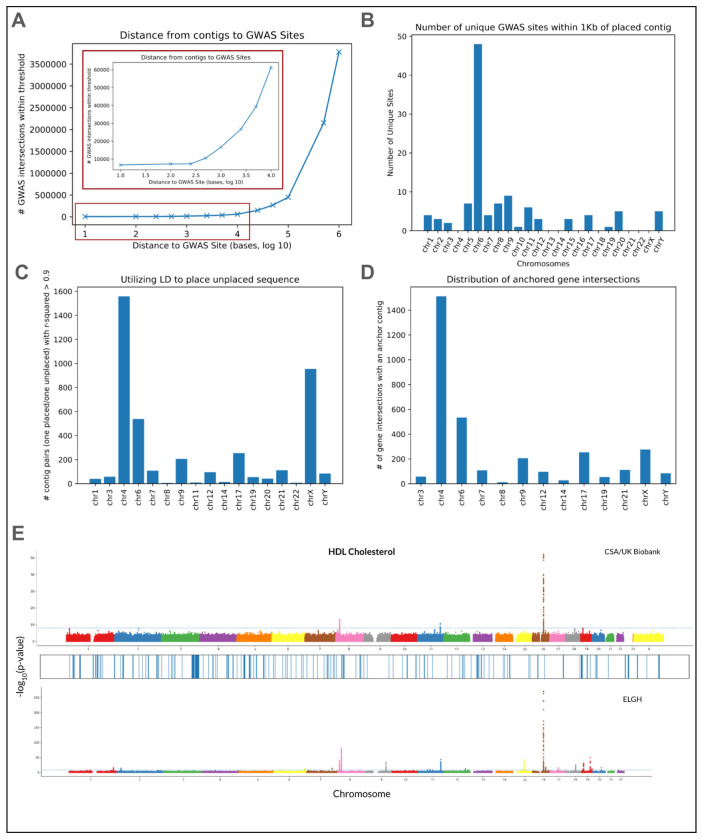
Functional Analysis. **(a)** The distance between a placed contig and a known GWAS site across a range of thresholds. **(b)** The number of unique GWAS sites with a placed contig within 1 kb across the 24 chromosomes. **(c)** Distribution of “anchored” contigs, where a pair of contigs have R^2^ score > 0.9, and one of the pair is placed and the other is unplaced. **(d)** Distribution of gene intersections with anchored contigs, where a pair of contigs have R^2^ score > 0.9, and one of the pair is placed and the other is unplaced. **(e)** Significant loci (p-value < 1e-8) associated with HDL cholesterol in the CSA population of the UK biobank (**top**) and the entire ELGH cohort (**bottom**), alongside locations of placed sequence against GRCh38 (**middle**).

The presence/absence of each shared contig across all 640 SAS individuals allowed us to measure linkage disequilibrium (LD) between pairs of contigs and identify “blocks” of placed contigs that likely belong to the same larger sequence. Specifically, we computed the squared Pearson correlation coefficient (r^2^) between all pairs of 13,875 shared contigs and found 644,578 pairs of contigs with r^2^ > 0.9 (0.33% of all pairs). Of these, 194,428 (0.1% of all pairs, 30% of pairs with r^2^ > 0.9) are between contigs on the same chromosome and 56,935 are between contigs within 10 kb of each other. We also observed 4,142 pairs where a placed contig is in strong LD with an unplaced contig (r^2^ > 0.9 across the 640 individuals). In these cases, the placed contig can act as an “anchor”, allowing us to narrow down the potential location of the unplaced contig within the genome. Within these anchor contigs, we identified 3,334 intersections with 24 genes on 13 chromosomes (Figure 5c–d), as well as 58 intersections with known GWAS hits.

#### Analysis of unplaced sequence

Despite placing approximately 20,000 contigs from the SAS set against T2T-CHM13, we assembled over 180,000 contigs that could not be placed. By querying this set of unplaced contigs using BLAST, we found that >90% had a high similarity match with non-reference human sequence or reference and non-reference primate sequences (Supplementary Table 2), supporting their non-contaminant human origin. There were also a small fraction of non-primate mammalian sequences with high similarity to these contigs, potentially reflecting highly conserved loci.

#### Analyzing pangenome placements

The limited tools available for pangenome analysis excluded the possibility of replicating the mate-pair based placements. Using only basic alignment to place contigs, we saw that contigs are aligned throughout both pangenome references, with ~8% of the assembled contigs (15,553/199,564) having an alignment length >500 bp; this followed the pattern seen with alignment-only placement against the T2T-CHM13 linear reference.

While the individual haplotypes used to construct the pangenome are annotated, there is no centralized set of annotations, GWAS catalog, or partner study identifying key loci in the added sequence. For these reasons, we stopped at basic placement against the pangenome reference and focused on the functional impact of sequence placed against linear references. In the future, we hope that this becomes an available method of analysis.

### Comparison to traditional variant finding approaches

We compared the sequence discovered through our “rescued read assembly” approach to conventional short-read-based SV callers. We first compare this sequence to the insertions found by two widely used variant discovery tools, Manta ^35^ and LUMPY ^36^. While both of these tools also call deletions (~6,000 per individual) which are generally not detectable through assembling unmapped reads into large contigs, we focused on comparing the called insertions to our assembled contigs from the 21 SAS individuals with long read validated data.

Manta identified an average of 2,300 insertions per individual, but only nine insertions per individual were longer than 500 bp (192 in total, with a maximum of 16 found in HG03898), and no insertions were longer than 1 kbp. In contrast, our approach assembled an average of 299 (SD = 9.3) contigs longer than 1 kbp per individual, an average of 36 of which could be placed against T2T-CHM13. The placed contigs overlapped some of the smaller and many of the larger (>500 bp) insertions called by Manta but also included hundreds of placed sequences that were not detected by Manta (Supplementary Figure 14–15), 85% of which were validated by long reads. LUMPY is largely focused on deletion calling and is limited in its ability to detect small insertions, but is able to detect large insertions through identification of breakends. Across the 21 SAS individuals, LUMPY detected none of these large insertions, despite our approach identifying and placing several large contigs in these individuals, as validated with long reads.

We also compared the sequence assembled by our approach to the sequences found by PopIns2 ^37^, which detects large non-reference sequences by assembling reads that do not have a high quality reference alignment. Across a subset of 10 1KGP individuals, one XX and one XY from each of the five 1KGP populations, PopIns2 assembled an average of 6,264 >1 Kb contigs per individual against T2T-CHM13. This is in line with the number of contigs and span of sequence our approach assembled when using poorly aligned reads (Supplementary Results, Supplementary Figure 16). Comparing the placement of these contigs against the contigs assembled and placed by our approach, we found that of the 267 contigs placed against these 10 individuals, 210 overlapped a placed contig from Popins2. However, our approach assembled more large contigs than PopIns2 (510 >5 Kb and and 95 >10 Kb contigs on average per individual from our approach vs 432 >5 Kb and 72 >10 Kb contigs from PopIns2) and also places a larger fraction of such contigs (10-12% of our contigs, vs 8% of the PopIns2 contigs) based on alignment, LD, and mapped mate pair reads.

### Functional analysis of hidden variation

#### RNA-seq analysis

We used RNA-seq data from the MAGE project ^38^, spanning 731 globally diverse individuals from 1KGP to evaluate the functional potential of the previously assembled contigs. We specifically focused on the 140 MAGE samples from SAS populations that overlap with our study. We aligned RNA-seq reads from each individual to their assembled unaligned read T2T-CHM13 contigs. Per individual, 800-1000 reads aligned to their contigs, with the majority spread across few contigs. While this is a small fraction of the input RNA-seq set (<0.1%), this is not unexpected, as the total length of assembled contigs per individual is <0.02% the size of the human genome. The majority of contigs with numerous RNA-seq alignments have BLAST hits to non-reference human and primate sequences, some of which is annotated as functional (Supplementary Table 3–4). These hits include annotated non-reference sequences from chromosomes 1, 2, 3, 7, 9, 15, X and Y.

We also aligned the RNA-Seq data from all 140 individuals against a combined set of filtered contigs from all 601 1KGP SAS individuals, and found 200 contigs with >100 read alignments and 19 contigs with >1000 read alignments (Figure 4a–b, Supplementary Figure 17–19), most of which BLAST showed had high similarity matches to non-reference human and primate sequences (Supplementary Tables 3–4). Across both these experiments, we found that the majority of highly aligned-to contigs appear in just one or a few individuals, with few classified as widely shared sequences (Figure 4f).

#### GWAS analysis

We next explored the functional potential of the assembled contigs by comparing the placed sequences against existing catalogs of trait-associated loci. We intersected the set of placed contigs against the T2T-CHM13 GWAS v1.0 catalog, and found 2,812 positions where a placed contig overlaps an annotated GWAS hit. These hits are spread across seven chromosomes: chromosome 6 (1,995), Y (601), 5 (208), 8 (5), 2, 9, and 17 (1 each). In addition, we identified >6,000 contigs within 100 bp of 36 unique GWAS hits and >15,000 contigs within 1 kbp of 112 unique GWAS hits (Figure 5a). We used long read data to validate the vast majority of the contigs placed within 100 bases (215/254, 84.6%) or 1 kb (506/587, 86.2%) of a GWAS hit in 21 SAS individuals (Supplementary Figure 20).

#### Comparisons of placements against loci associated with biomarker traits

Finally, we investigated if any of the placed contigs overlapped with or were near trait associated loci from the UK Biobank (UKB) ^39,40^ and East London Genes & Health (ELGH) study ^41^. The former contains ~400,000 individuals of all ancestries, and the latter contains data from ~37,000 SAS individuals in the UK.

Across the 21 biomarker traits from the Pan-UK Biobank ^42^ and 42 biomarker traits from the the Genes and Health (ELGH) ^41^ dataset, we saw only a handful of placed contigs within 10 Kbp of significant locus at a p-value threshold of 1 × 10^−6^. We found more placements within 1 Mbp of a significant locus, with loci associated with 8-14 traits close to placed contigs as we varied the p-value threshold from 1 × 10^−8^ to 1 × 10^−6^ respectively (Supplementary Table 5). However, we did not find any placements within 1 Mbp of a significant locus within just the smaller UK Biobank “CSA” (Central/South Asian) ancestry group (n = ~7,000) until we lowered our threshold to 1 × 10^−5^. However, as ~90% of our assembled contigs remain unplaced, there may still be contigs associated with these traits that cannot be identified simply by looking at nearby placements.

## Discussion

We investigated the diversity present in South Asian populations by aligning high-coverage short read sequencing data from 640 individuals across 24 populations to reference genomes and assembling the unaligned reads. These assembled contigs contain variant sequences whose functional impact we then attempted to ascertain through placement, RNA-seq analysis, and comparison to existing nucleotide databases and biobanks.

We found that complete reference genomes enable better alignment, as reflected in improved alignment rates and smaller amounts of sequence assembled from unaligned reads. Despite these improvements, we still assembled hundreds of megabases of sequence (tens of megabases of non-redundant sequence), much of which cannot be placed against the reference but which we demonstrate is probably of human origin. Within the thousands of placed sequences, there are thousands of overlaps with genes and GWAS hits. We used RNA-seq data to directly identify hundreds of contigs with functional potential and long read data to validate >80% of the assembled contigs and >85% of the gene and GWAS hit overlaps. We compared this assembled sequence to sequences from non-SAS populations to understand how this variation is distributed within and across populations, and we compared our results to those from existing SV calling tools to show how much of this sequence is undiscovered or discarded by conventional methods.

While these sequences are assembled from variation in a South Asian cohort, they are typically not strongly enriched in frequency within South Asian populations. Rather, most of these variant sequences are either globally common or rare even within the South Asian populations^43^. The fact that this much global variation is discoverable from this South Asian cohort highlights how much genetic variation remains undiscovered and the importance of expanding the scope of population genetic studies beyond linear reference genomes.

Further improvements in reference genomes, especially the expansion of current pangenome builds with more high quality genomes, will help to capture more diversity. The generation of more high quality DNA sequencing will further expand the amount of variant sequence we can find, and new auxiliary data will help to validate these sequences and understand their functional potential ^44^. This approach can also be applied to existing sequencing data from populations around the world to discover previously missing variation, including in groups that have been traditionally underrepresented in genomics ^45–48^.

Our analysis provides a model for expanding beyond a single reference genome to more comprehensively characterize human genetic variation, establishing reference resources for the benefit of all human populations.

## Methods

### Data

We used read data from the 1000 Genomes project (1KGP) ^21^ and the Simons Genome Diversity Project (SGDP) ^22^, totaling 640 individuals from 24 South Asian populations across the two sets. The read data from these sets is high coverage (>30x), high quality, short paired reads. The 601 SAS individuals present in the 1KGP set are from five South Asian populations. Three are in the South Asian diaspora - Gujarati in Houston (GIH), Sri Lanka Tamil in the UK (STU) and Indian Telegu in the UK (ITU) - and two are in South Asia itself - Punjabi in Lahore (PJL) and Bengali in Bangladesh (BEB). The 601 individuals are largely evenly distributed between the five populations, with 109-144 individuals from each. The SGDP set contributes 39 individuals, spread across 19 different groups within India, Pakistan, Nepal and Bangladesh. Most of these are in pairs from each group, with a single individual from one population (Khonda-Dora from India) and four from another (Punjabi from Pakistan) the only exceptions.

These reads are aligned against two linear references and two pangenome builds. The two linear references are GRCh38 ^24^ and T2T-CHM13 v2.0 ^3,14^. We used the Minigraph v1.0 and Minigraph-Cactus v1.0 builds of the newly released Human Pangenome Reference Consortium (HPRC) draft pangenome references. Both pangenome references are constructed over the T2T-CHM13 reference and include variation present in 47 selected individuals from the 1KGP set, one of which is a SAS individual present in our set. The Minigraph reference contains only SVs larger than >50bp, while the Minigraph-Cactus reference is further augmented to include base-level alignments for other variants.

We performed validation of our assembled contigs using long read data from the 1KGP ONT effort ^23^, which is available for 21 SAS individuals in our 1KGP set. Our functional analysis was augmented using RNA-Seq data from the MAGE dataset ^38^, which provides RNA-seq data for 140 SAS individuals. For our GWAS and Biobank analysis, we used the standard T2T-CHM13 GWAS catalog and the phenotype manifests from the Pan-UK Biobank ^42^ and the East London Genes and Health effort ^41^, focusing on the quantitative biomarker traits in each.

More details about the data characteristics of the read data and its alignments to the linear references can be found in Supplementary Figure 21, Supplementary Figure 22, Supplementary Figure 23 and Supplementary Figure 24.

### Read alignment and extraction

Reads from the 640 SAS individuals in the 1KGP and SGDP sets were aligned to GRCh38 and T2T-CHM13 using bowtie2 v2.4.1 ^49^, and the unaligned and poorly aligned (quality value < 20) reads were extracted using samtools v1.14 ^50^. Unaligned reads of two forms are extracted from these alignments: both reads where the read and its pair are both unaligned, as well as single unaligned reads with a mapped mate read (Supplementary Note 1).

### Assembling unaligned reads

We tested three assemblers on a selected group of 10 SAS individuals (two from each of the 1KGP populations): SPAdes v3.15.3 ^51^, MaSuRCA v4.0.9 ^52^ and MEGAHIT v1.2.9 ^25^. There was some variation in the number of short contigs (SPADes produces far more than the other two), but the number of large non-contaminant contigs varies by no more than 5% between assemblers. All assemblers were tested using their default parameters, with the four sets of unaligned reads extracted in the previous step passed in as input. The biggest difference between these assemblers is the variation in runtime. We opted for the fastest of these three assemblers - MEGAHIT - in an effort to speed up analysis and reduce costs (Supplementary Note 2).

### Filtering assembled sequence

As in the APG analysis, we first discarded all contigs less than 1 Kb in size. This was done by filtering the assembly FASTA file for sequences longer than the target length, with all other sequences being discarded (Supplementary Note 2).

We also screened these sequences for contaminants using two tools: Centrifuge v1.0.3 ^27^ and BLAST ^26^. For Centrifuge, all assembled sequences were compared against the “Bacteria, Archaea, Viruses, Human” database, and sequences categorized as non-human are removed. With BLAST, we used the “refseq_genomic” database, and removed all sequences that have a high scoring match to a non-human sequence. The contaminant sequences can be identified by parsing the resulting outputs, and these sequences were then cut from the FASTA file. After the filtering stage, we were left with a list of >1 Kb, non-contaminant contigs from each of the SAS individuals for use in the later stages (Supplementary Note 2).

### Assembly of non-SAS sequences

We selected 10 individuals - 5 XX and 5 XY - from each of the 21 non-SAS 1KGP populations. This was done by iterating through a list of participants sorted by participant ID, and choosing the first 5 XX and 5 XY from each population. As done with the SAS set, these individuals’ reads were then aligned against T2T-CHM13 using bowtie2.4.1, the unaligned and poorly aligned reads were extracted, the reads were assembled using MEGAHIT with default parameters, and filtered for size and contaminants.

### Reference vs reference comparison

Another analysis we performed to highlight the improvements made in T2T-CHM13 over the previous GRCh38 reference is “cross-alignment”. Contigs assembled from unaligned reads against T2T-CHM13 were aligned against those assembled from unaligned reads against GRCh38, and vice versa. We also aligned the unaligned contigs from both references against both references, to demonstrate how T2T-CHM13 is more effective at “rescuing” these contigs. These alignments were done using Minimap2 v2.22 ^32^, and the number of contigs that align or fail to align from and against each reference is noted.

### Categorizing sequence as shared or unique

We evaluated how much of the sequence in the T2T-CHM13 unaligned contigs was unique to the individual it is present in and how much was present in multiple individuals using all-vs-all alignments of the contigs to themselves.

The all-vs-all alignment was performed using Minimap2 v2.22 ^32^, and the resulting output file was used to collapse the shared sequence. To do this, we repeatedly iterate through the output of the all-vs-all alignment and consider contigs with alignment lengths to each other greater than 90% of their own length to be the same sequence. We selected the larger of these sequences to be the representative “super-contig”, and we noted the other sequence as being equivalent to or is derived from this one. We repeated this process until a full iteration through the set occurs without any more sequences being collapsed down, leaving a finalized list of “super-contigs”, a list of which contigs have collapsed into each super-contig, and a list of sequences that have undergone no collapsing and are therefore unique. Combining the list of super-contigs with all non-collapsed sequences gave us our finalized, “non-redundant” set of SAS contigs (Supplementary Note 8).

This approach acts as a floor on the amount of shared sequence, as it only merges contigs that are almost entirely identical. However, this approach does not consider contigs that share small subsequences or overlap only slightly at the ends. These situations would be best captured through all-vs-all comparisons and collapsing with no threshold for minimum alignment length. Alternatively, using a similarity metric such as Mash distance ^53^ could quickly estimate the similarity between contigs and decide which ones to merge. Such an approach would account for the case where two contigs have smaller regions of similarity dispersed throughout their length. For Mash distance calculations we used Mash v2.3 ^53^ with default parameters (1000 hashes, sketch and compute overlap in one step) on the Cartesian product of the set of individuals and themselves.

### Assessing non-SAS contribution to SAS contigs

For each of the 210 non-SAS individuals, consisting of 5 XX and 5 XY individuals from the 21 non-SAS 1KGP populations, we aligned their reads using bowtie2 v2.4.1 to the finalized non-redundant set of SAS contigs. We then parsed the alignment output logs to extract their alignment rates, and then further analyzed these counts to generate sub- and super-population summaries.

### Placing assembled sequence

In order to place the contigs against the reference, we followed two approaches: direct alignment of the contigs using Minimap2, or using mate-pair reads that have been mapped to the reference.

For direct alignment, we aligned the contigs against the chosen reference, and reported the locations of all contigs with alignments over >500bp. This alignment and filtering stage was performed using Minimap2 and then a simple post-alignment filtering based on the length of the alignment, which is stored in one column of the output .paf file (Supplementary Note 3).

The mate-pair placement method is the approach taken in the construction of the African Pangenome ^15^. During our read extraction step, we explicitly extracted unaligned reads that had mapped mates, and once we knew which contig an unaligned read was assembled into, we could use the mapped mate to anchor the entire contig (Supplementary Note 3).

#### Intersections with and analysis of annotated elements

We used the “JHU RefSeqv110 + Liftoff v5.1” annotations generated by the T2T consortium and intersected this with the list of placements generated in the previous step. We computed these intersections using bedtools v2.30.0 ^34^, using the “-wb” parameter to output the details of the intersection annotated element. The bedtools output could then be further analyzed using Python scripts to generate counts for the number of placements per gene, the number of times a gene is intersected with, and other such counts (Supplementary Note 4).

We obtained details on the gene types, status, summaries and expressions from their NCBI pages. This can be done using the NCBI E-utilities or simply by scraping the corresponding webpage for each affected gene - we primarily used the latter, due to technical issues with the E-utilities tool. The vast majority of the summary and expression data come from RefSeq and “HPA RNA-seq normal tissues” project ^54^.

#### GWAS intersections with placed contigs

We used the same steps as the annotated region intersections, except this time using the “GWAS v1.0” annotations generated by the T2T consortium. We used “bedtools intersect” with the same configuration to generate the list of all direct overlaps between GWAS elements and placed contigs (Supplementary Note 4).

In order to account for placements that are close to but not strictly overlapping the GWAS elements, we implemented a Python script to compare the coordinates of placed contigs against those of known GWAS elements, and compute the distance between all placed contigs in a chromosome and all its GWAS sites. This was done by computing the distance between all GWAS sites and the start and end points of each contig, thus allowing us to both identify all overlaps but also pre-compute all distances between all pairs of contigs and GWAS sites. We could then alter the threshold we want to test for, and count the number of interactions with a distance lower than this threshold.

### Analysis of unplaced sequence

We queried the unplaced sequences against the BLAST “nt” database to ascertain their potential significance, and extracted the top 50 highest scoring hits for each contig. We did this by parsing the BLAST output to extract the lines containing the top matches after each query sequence, before tallying up the number of sequences included in those “top hits”, and outputting these to a table (Supplementary Note 6).

### Linkage disequilibrium of shared sequence

From the 13,875 sequences present in multiple individuals, we generated a 640 x 13,875 binary grid representing the presence (1) or absence (0) of each of the shared sequences (or those that have been collapsed down into them) in each of the 640 SAS individuals. Each column in the grid represents the presence or absence of a given contig in each of the 640 individuals in the SAS set, and two contigs that are in LD with each other will have highly similar columns.

We computed a pairwise r^2^ between each of the 13,875 contigs, and then filtered out pairs that had a score higher than our chosen threshold. We then used our placement data to identify pairs of contigs placed within the same chromosome or within a threshold distance, and to identify pairs where one contig is placed and the other is not.

### Pangenome analysis

We tested a range of pangenome aligners, including GraphAligner ^55^, VG ^56^, vg giraffe ^57^, Minigraph ^58^ and Minigraph-Cactus ^59^. We found the vg suites of tools to be easiest to use, but the outputs across the aligners are largely consistent. Regardless of tool, we either forced the output of the aligners to be in the GAM format, or used existing conversion tools to create GAM files. These GAM files can then be converted to the standard SAM/BAM/CRAM format for read extraction (Supplementary Note 9). Once in the SAM format we continued to filter and extract reads exactly as we did against the linear references, and repeat the same assembly and filtering steps for these contigs.

Placement was also more complicated due to there being multiple parallel variant sequences, differences in coordinate systems and indexing between aligners (or even different versions of the same aligner), and the increased computational complexity of now performing alignment against a branching sequence graph. We obtained basic coordinates and counts from parsing the various alignment files generated by the different aligners, all of which are run with the finalized list of contigs and the pangenome reference as input and default parameters.

Once placed, there are few resources (GWAS catalogs, annotations, identified significant loci) that have been fully translated into the new coordinates and formats used in these pangenome builds. Therefore, we did not replicate this analysis against the pangenome references.

### Long read validation

We used long read data for 21 SAS individuals from the 1KGP ONT effort to validate our contigs ^23^. We first construct basic long read assemblies for each of the 21 individuals. This was computed using the Flye assembler ^33^, with default Flye v2.8.1 parameters for “raw-ONT” reads, and with just the long read sets as input (Supplementary Note 7).

The previously unaligned short reads were then aligned against these assemblies using bowtie2, and the assembled unaligned read contigs can be aligned using Minimap2 (Supplementary Note 9). We used the same tool versions (bowtie2 v2.4.1, and Minimap2 v2.22) and parameters for each of these as the read alignment and contig mapping stages in the analysis of the short read linear reference data.

For short reads, we filtered the resulting SAM alignment file to obtain a list of the reads that now align as well as a list of the reads that remain unaligned. Similarly, the output of the Minimap2 alignment of contigs to this individual-specific long read reference was filtered based on the alignment quality and length.

#### Long read validation of contig placements

We overlapped the list of contigs with high quality alignments in each of the 21 individuals against the list of placed contigs and their intersections with gene and GWAS elements, giving us a list of long read validated intersections. This was done by iterating through the list of placements that intersect an annotated element or GWAS site, and checking if the placed contig had an alignment to the long read assembly.

### RNA-Seq analysis

We used STAR v2.7.10 ^60^ to align the RNA-Seq data obtained from the MAGE dataset ^38^ from each of the 140 individuals against the short read contigs assembled from their unaligned reads against T2T-CHM13, and selected all contigs with large numbers of RNA-Seq alignments (Supplementary Note 5).

We tested both single and two-pass alignments, and found little to no changes between these two approaches. Alignment was also tested without splice junctions or annotations, and with a list of known human annotations; once again there was no significant difference. The remaining numerical and memory parameters (including “genomeSAindexNbases” and “limitGenomeGenerateRAM”) were left as default, or amended to STAR’s recommendation (Supplementary Note 5).

Once we had identified the most aligned-to contigs, we used the BLAST “nt” database to query for these sequences and report all top hits. As we did for the analysis of unplaced sequence, we parsed the BLAST output for each query sequence and output the top 50 highest scoring hits for each contig (Supplementary Note 6).

We also performed an exhaustive all-vs-all alignment of all the RNA-Seq reads across the 140 individuals against all the contigs assembled across the 640 SAS individuals. The parameters for this alignment were slightly adjusted, mainly so that STAR can handle the sharp increase in the number of reads (notably, the “genomeSAindexNbases” and “limitGenomeGenerateRAM” are adjusted as per STAR’s requirements). Similar to the previous analysis, we extracted the most aligned to contigs (>200 RNA-Seq alignments per contig), and ran them through the same BLAST query process. The top 50 BLAST hits (if there are that many) for each contig were then extracted.

### Biobank analysis

We used the UK Biobank Phenotype Manifest from the Pan-UK Biobank effort ^42^ to obtain our chosen set of 21 quantitative biomarker traits. Using the provided data for each trait, we were able to extract a list of all chosen loci in the genome and their potential association with each of the traits. The manifest represents this association as the negative log of the p-value, and this p-value is computed for both the entire UK Biobank set and for each of the individual ancestry groups within the Biobank. For our analysis, we used the p-values across the “CSA” ancestry group and the entire UK Biobank set. The Genes and Health dataset ^41^ is formatted in a similar way, and contains the associations between all chosen loci in the genome and 42 quantitative biomarker traits.

For each trait, we then “swept” across all potential loci, and identified all loci that have significance above our chosen p-value threshold. For all significant loci within 50 Kb of each other we selected the highest scoring loci (i.e. the one with the most significant p-value) as the representative “peak” for the entire group. We computed the pairwise distance between all selected peaks for a given trait and all placed contigs across the SAS set, and identified all peak/placed contig pairs that are within 1 Mb of each other. This process is repeated for all 21 biomarker traits in the UK Biobank, and for the 42 quantitative biomarker traits in the Genes and Health dataset.

### Comparison to existing variant discovery tools

We compared the assembled contigs from 21 SAS 1KGP individuals with LR data to the variant calls made in those individuals using Manta ^35^ and Lumpy ^36^. Both tools were run with default parameters using the output of the alignment stage (Supplementary Note 10). Manta uses the .cram file from the alignment stage directly, while there is an initial preprocessing step to extract the split and discordant reads for Lumpy.

Both tools output a vcf file that detailed the structural variants they find, which we parsed to determine the location, type (“INS” for insertions, “BND” for breakends of large insertions in Lumpy) and size of the SVs. We then compared this against the list of contigs assembled from these individuals, the contig placements in this set, and which of the contigs were validated with long reads.

For PopIns2 ^37^, we selected 10 samples from the 1KGP SAS set, and followed the steps outlined in the PopIns2 Github repository using default parameters, starting from the alignments of these individuals’ reads against T2T-CHM13 (Supplementary Note 10). We then parsed the resulting assembly and vcf files to compare against the assembled and placed sequence from our approach.

## Supplementary Material

Supplement 1

Supplement 2

## Figures and Tables

**Figure 1.**
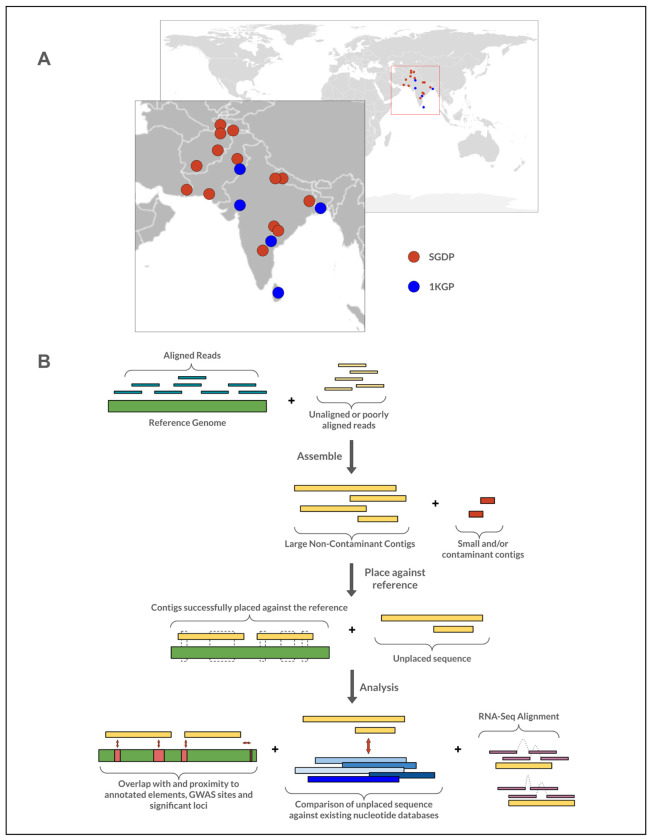
Input Data and “Read Rescuing” Pipeline. **(a)** The source of the data we are using; red dots are samples from SGDP, blue dots are from 1KGP. **(b)** A brief overview of our pipeline. Existing short read data is aligned against a chosen reference, after which the unaligned reads are extracted and assembled into larger contigs, which in turn are compared against the reference genome. Successfully placed contigs are checked for overlaps with and proximity to known annotated elements, GWAS sites, and loci significantly associated with traits and conditions; unplaced loci are compared to existing nucleotide databases. A full pipeline overview is available in Supplementary Figure 2.

## Data Availability

Our analysis was performed on both our local computing cluster at JHU and on the NHGRI AnVIL cloud platform, with the initial experiments performed on the former and the full set being analyzed on the latter. Experiments on AnVIL were run with a number of Docker environments and using WDL workflows. The analyses were computed using a range of established bioinformatics tools and custom scripts, and plotting was performed using existing Python libraries, such as sklearn ^61^ or matplotlib ^62^. Each tool used has been mentioned in the relevant methods section, and all relevant scripts and documentation can be found at github.com/arun96/SouthAsianGenomeDiversity. The AnVIL workspace used for this analysis can be found at https://anvil.terra.bio/#workspaces/anvil-dash-research/south-asian-genome.
